# The Uprising of Mitochondrial DNA Biomarker in Cancer

**DOI:** 10.1155/2021/7675269

**Published:** 2021-07-15

**Authors:** Siti Zulaikha Nashwa Mohd Khair, Siti Muslihah Abd Radzak, Abdul Aziz Mohamed Yusoff

**Affiliations:** Department of Neurosciences, School of Medical Sciences, Universiti Sains Malaysia, Health Campus, 16150 Kubang Kerian, Kelantan, Malaysia

## Abstract

Cancer is a heterogeneous group of diseases, the progression of which demands an accumulation of genetic mutations and epigenetic alterations of the human nuclear genome or possibly in the mitochondrial genome as well. Despite modern diagnostic and therapeutic approaches to battle cancer, there are still serious concerns about the increase in death from cancer globally. Recently, a growing number of researchers have extensively focused on the burgeoning area of biomarkers development research, especially in noninvasive early cancer detection. Intergenomic cross talk has triggered researchers to expand their studies from nuclear genome-based cancer researches, shifting into the mitochondria-mediated associations with carcinogenesis. Thus, it leads to the discoveries of established and potential mitochondrial biomarkers with high specificity and sensitivity. The research field of mitochondrial DNA (mtDNA) biomarkers has the great potential to confer vast benefits for cancer therapeutics and patients in the future. This review seeks to summarize the comprehensive insights of nuclear genome cancer biomarkers and their usage in clinical practices, the intergenomic cross talk researches that linked mitochondrial dysfunction to carcinogenesis, and the current progress of mitochondrial cancer biomarker studies and development.

## 1. Introduction

In the new era of medicine through modern research and technology, advances in predictive diagnostic and precision medicine can lead to powerful discoveries and the most effective treatments for the patients. The biomarker is the primary choice for clinical trial implementation due to its reliability and beneficial purposes, especially in cancer research. All human cancers arise from abnormal cells' uncontrollable proliferation due to an enabling characteristic, genomic instability [1, 2]. This characteristic is needed by cancer cells in order to acquire functional capabilities to survive, proliferate, and circulate [2]. However, the exact stage of genetic and molecular changes that occur during cancer development remains unresolved [3].

Genomic maintenance systems possess the ability to spot and repair any DNA defects in retaining a low mutation rate in each cell generation. Meanwhile, cancer cells frequently increase the rates of mutation that orchestrate tumorigenesis [2, 3]. It has been proposed that many forms of genomic instability are the culprit underlying certain carcinogenesis. Chromosomal instability (CIN) was initially proposed as one of the most frequent changes observed in cancer cells which often results from aberrations in chromosome structures and numbers [3–5].

Over the past decades, enormous progress in epigenetics and nuclear genome-based studies has been achieved in improving the conservative cancer screening methods by finding specific markers. Due to multiple genomic alterations observed in cancer cell progression, researchers intended to find new options, besides the two fundamental studies involving genetic mutations and epigenetic modification in nuclear genome. Efforts have been focused on the mitochondrial genome, a widely known nuclear genome codependent in several mechanisms such as replication and repair [6]. Moreover, it has been described that nuclear genome expression is highly responsive to the mitochondrial respiratory functions through a process called mitochondria–nucleus retrograde signaling. These close interconnections suggest essential characteristics for intracellular and extracellular homeostatic adaptation [6, 7].

In this review, we will extensively describe the history of biomarker and carcinogenesis, which will be followed by detailed reviews of a research turning point from nuclear to mitochondria, leading to the discovery of the mitochondrial DNA (mtDNA) alterations as established and potential biomarkers for cancer. New information on current findings provided by this review will give clear insights for noninvasive early cancer detection, thus holding future advancement in new cancer therapeutics.

## 2. Biomarkers Up Close

Studies of human diseases recall all types of biomarkers used by generations of scientists, physicians, and epidemiologists [8]. Its early efficacy and safety evaluations are the main points of establishing “proof of concept” either in tissue sample (in vitro) or in animal model (in vivo) studies [9]. According to the definition proposed by the International Program of Chemical Safety led by World Health Organization (WHO), the biomarker is described as any substance, structure, or process that is measurable in the body or its product that can influence or predict the incidence and outcome of a disease [10].

According to Roméo et al., there were three classifications of biomarkers: exposure, effect, and susceptibility [11]. Biomarker of exposure is referred to as the measurement of exogenous chemicals or their metabolites within an organism, besides measuring the interaction results between a xenobiotic compound and some target molecules or cells [12]. Another closely related biomarker is the biomarker of effect. Defined as an alteration of endogenous factors caused by exposure towards an exogenous agent, it is very useful in hazard identification, toxic agents screening, and toxicity characterization process [13, 14]. The third type is biomarker of susceptibility, which referred to the genetic polymorphism predisposition of individuals and their external multifactorial influencers. Able to initiate various types of biological responses towards exogenous agents, multiple external factors such as age, diet, ethnicity, and health status are influencers determining varies responsiveness in individuals [14].

Surrogate endpoints (indirect measures) are often used to substitute clinical endpoints (feelings, functions, and survival of patients) but with proper validation done beforehand [13, 15]. It is believed that all surrogate endpoints are biomarkers, but only a few biomarkers could reach the standard of becoming a surrogate endpoint. They were expected to scientifically predict clinical outcomes such as benefits or risks or the lacking of both. Additionally, there is a high probability that the same biomarker will be introduced to clinical practice with similar disease response measurements [9]. For example, measuring blood pressure as a surrogate endpoint is highly predictive for effects on stroke and moderate for prediction of cardiovascular death and overall mortality, while inefficiently predictive for heart failure effects [16]. Several values are the reasons that clinical practitioners chose biomarkers as surrogate endpoints. They allow affordable trials and shorter time consumption to observe the intervention effects with multiple endpoints of observation options that require smaller sample size. Thus, these likely elevate the reliability and effectiveness of data collecting, in compliance with easily quantified surrogates (laboratory measurements or imaging biomarkers) [17].

Historically, biomarkers were simultaneously used for biological and health monitoring [18]. Biomarker development for early detection has always been the top priority but inevitably challenging in the cancer research field [19]. Cancer-specific marker is considered faultless if positive result (individuals with positive marker) indicates 100% sensitivity and elevated marker (only in cancer patients) shows 100% specificity [20]. In 1965, Dr. Joseph Gold established the first approved test by discovering a fetal tissue substance, the carcinoembryonic antigen (CEA), in the blood of colon cancer patients [21]. During the 1980s, numerous biomarkers for different cancers have been discovered, for example, CA-125 for ovarian cancer. Even so, these markers were not single-cancer-specific, but their reliability as early disease indicators was proven [20].

Biomarkers can be utilized as a screening tool for an early indicator of malignancy-risk development which beneficially enables early intervention and prevention. The possibilities are higher with the advancement in genetic testing via the findings of hereditary cancer-susceptible genes [19]. They are also advantageous diagnostic aids, involving patients with symptoms. Serum diagnostic biomarkers are inefficient for early cancer diagnosis due to its lack of sensitivity and specificity but were found useful in diagnosing both benign and malignant tumors [19]. Meanwhile, prognostic biomarkers are vital in identifying patients with the clinical event, disease recurrence, or progression [22]. On the other hand, predictive biomarkers are known to identify the sensitivity and/or resistance of cancer patients towards specific agents or medical product exposure [22, 23]. Prediction of the outcomes was carried out based on the preferential effects, which allow the researcher to determine the rate of therapy interventions' effectiveness by comparing the investigational therapy group with the control group [24].

## 3. From Nuclear to Mitochondrial

### 3.1. Intergenomic Cross Talk

The intergenomic communication between the nucleus and mitochondria is likely to happen bidirectionally. It creates a linkage to connect the extensive prevalence of somatic mtDNA mutations and mitochondrial dysfunction with various cancer and progression studies. After experimenting with different human cancer cells, the nuclear genome seems to have experienced alterations caused by mtDNA depletion and mutations [6]. The dual genome bidirectional cross talk was reported by Ma and colleagues, who measured *p53* gene expression––the responsible gene for energy metabolism and tumor suppression. The nuclear gene's effect was excluded from the study using transmitochondrial cybrids, to purely investigate the outcome of mitochondrial dysfunction in cancer. The genetic and functions of mitochondria were altered in cancer cells, thus sending the signals to the nucleus and regulating *p53* expression [25].

**Figure 1:**
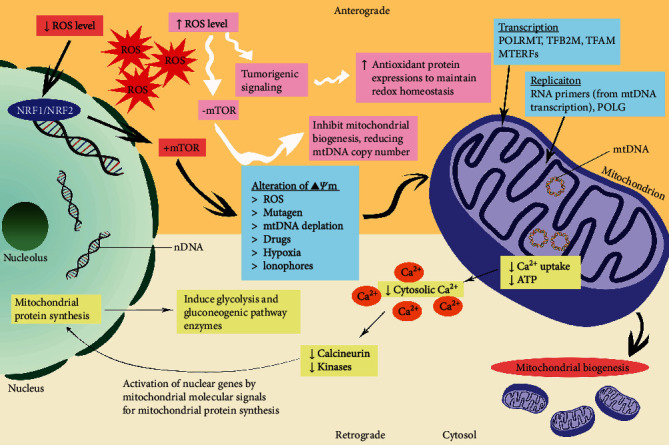
Illustration of some pathways involved in intergenomic cross talk between nucleus and mitochondrion.

A few studies suggested that mtDNA depletion plays a crucial role in triggering the intergenomic cross talk between mitochondria and the nucleus, subsequently contributing to tumorigenesis [26, 27]. Proving the link, Clayton had stated that mtDNA replication proteins are nuclear-encoded [28]. As suggested by Wallace, about 1500 nDNA encoded mitochondrial genes [29]. The communication was achieved by intergenomic cross talk, a vital communication system that regulates mitochondrial protein synthesis, subsequently used in mitochondrial biogenesis process. Besides, the cross talk is also important for activating viable responses to cope with mitochondrial dysfunctions [30–32]. Several pathways are involved in the communication system involving the nucleus–mitochondria (anterograde signaling pathway), mitochondria–nucleus (retrograde signaling pathway), and the pathways in between [33, 34]. Some of the pathways involved in intergenomic cross talk between the nucleus and mitochondrion are illustrated in Figure 1.

#### 3.1.1. Anterograde Signaling Pathway

Evolved symbiotic relationship between mitochondria (free-living bacteria) and eukaryotic cells suggested the merging of nuclear-encoded glycolysis pathway and cytosolic components with the protomitochondrial oxidative system [35]. The new complex relationship had most of the mtDNAs transferred to the nuclear genome. Hypothetically, nDNA is now responsible for encoding respective genes vital in cellular morphology and physiology, other than glycolytic and oxidative metabolism [29, 35]. The interconnection response is able to regulate mitochondrial proteome by inducing the expression of nuclear-encoded mitochondrial genes [36].

The anterograde signaling is predominantly a process of nucleus controlling gene transcription and cytoplasmic mRNA translation, responsively to external signals which regulate OXPHOS and mitochondrial biogenesis [31, 32, 36]. In other words, the pathway is viable to ensure the sustainability of mitochondrial bioenergetics and dynamics. Besides, it monitors the content of healthy mitochondria by regulating several components such as copy number and mitochondrial activity. Consequently, important mitochondrial homeostatic events such as mitophagy, fusion or fission, and biogenesis were triggered by anterograde pathway signals, as well as promoting cell growth and survival when necessary [37–39]. As a responsible pathway for homeostasis adaptation, the anterograde signaling pathway can detect any nDNA damage or nuclear stress. Hence, signal transduction to mitochondria occurred that regulated mitochondrial bioenergetic by reducing its metabolism [36].

Mitochondrial biogenesis is a complex process which increases the coordination of mitochondrial mass and bioenergetic capacity when triggered [39]. Temporal and spatial cues such as nutritional abundance or deprivation, temperature, and hormonal alteration are likely to orchestrate anterograde pathways (carbon and nitrogen sources sensitive pathways) for gene expression regulation [40, 41]. Therefore, biogenesis triggered transcriptional regulatory proteins which initiates nuclear-encoded transcription [42]. This includes the several crucial nuclear-encoded components are such as POLRMT (RNA polymerase), TFB2M (transcription initiation factor), TFAM (transcriptional stimulatory factor), and MTERFs (termination factors) for mtDNA transcription [28, 39]. On the other hand, mtDNA replication was also initiated by biogenesis. RNA primers produced from mtDNA polymerase gamma (POLG) activity during transcription process were used to enhance mtDNA copy number [39, 43].

According to Scarpulla, the expression of mitochondrial respiratory chain and basal transcription components were controlled by indirect regulators known as nuclear respiratory factors (NRF1 and NRF2) [44]. However, direct regulation occurred when these regulators were imported into the mitochondria, becoming the origin of transcription alteration. Other examples of direct regulators for nuclear-encoded mitochondrial gene expression are *p43* (T_3_ receptor) and *p53* tumor suppressor [42].

#### 3.1.2. Mitochondrial Retrograde Signaling and Response

The linking bridge between mitochondria–nucleus is known as retrograde signaling, a triggered pathway due to mitochondrial dysfunction or loss of mitochondrial membrane potential (*Δψ*m). Subsequently, it induces communication with the nuclear genetic compartment for homeostatic purposes [6, 31]. Therefore, changes in cellular metabolic and functional state triggered nuclear gene expression profile, cell morphology, and physiology modifications [31]. Knowingly, the retrograde signaling pathway is able to extend the cell's replicative life span by engaging a group of signal transduction proteins [45]. The retrograde signaling pathway was firstly discovered in mtDNA-lacking yeast petite cells, *Saccharomyces cerevisiae*, by Liao and Butow [46]. Miceli et al. suggested that retrograde signaling was initiated by ATP concentration reduction, resulting from the disruption of the respiratory chain [47]. However, the exact nature of the pathways involved was not well understood due to the pleiotropic characteristic of the retrograde pathway [36].

Pleiotropic features allowed mammals' retrograde pathway to be characterized based on pathway in yeast colonies, as their close resemblances include several parallel regulatory events, despite of different microenvironment and cells [48]. The retrograde response pathways are further divided according to various triggers: energetic stress, calcium-dependent, and reactive oxygen species (ROS) [36]. Initially, retrograde signaling pathway response towards energetic stress was observed in yeast, regulating carbon and nitrogen metabolism for sustaining a balance mitochondrial redox state [30, 36, 37, 49, 50]. The peroxisomal citrate synthase (CIT2), a gene involved in the glyoxylate cycle [51], was firstly discovered to be a retrograde response mediator by Liao and colleagues [52].

The intracellular Ca^2+^ regulation is vital for mitochondria via its close interaction with the endoplasmic reticulum [53]. It was suggested that the retrograde responses are affected by metabolic cues or alteration in mitochondria-related intracellular Ca^2+^ [7]. A work conducted in 2002 proposed that cancer progression and metastasis are influenced by the activation of the retrograde signaling pathway when cytosolic Ca^2+^ elevates [54]. Increment occurred due to *Δψ*m disruption, which consequently abrupt mitochondrial ability for Ca^2+^ uptake [7, 54]. There are two sources of Ca^2+^ regulation for retrograde signaling pathway, one, Ca^2+^/calcineurin-mediated for translocating nuclear transcription factors into the nucleus. This promotes protein synthesis that induces glycolysis and gluconeogenic pathway enzymes [32, 54]. Another pathway involves Ca^2+^-dependent mitogen-activated protein kinases' direct activation, particularly causing transcription factors stimulation (Figure 1) [32, 36].

According to several sources, redox activity regulation increased stress resistance; thus, signal transduction from ROS activated retrograde pathway [55, 56]. ROS are mainly produced by mitochondrial electron transport chain (ETC) during aerobic metabolism. Reducing ETC/OXPHOS capacity leads to cellular energy deprivation which released stress signals. Therefore, the regulation of mitochondrial ROS production rate and activity occurred, affecting mitochondrial ROS signaling and redox-related events [7, 57]. Retrograde signaling pathway is also known to be interconnected with few other metabolic stress responsive pathways such as ceramide signaling and mammalian target of rapamycin (mTOR) [31, 45]. The rate of ROS production from ETC influenced mTOR––the major regulator of nuclear protein synthesis, which indirectly affected mitochondrial biogenesis [58]. Uncompromised antioxidant systems enable ROS to exceed rate detection that switches off mTOR pathway and inhibiting mitochondrial biogenesis [33, 37, 59].

## 4. Mitochondria and Cancer

### 4.1. Human Mitochondria and Its mtDNA

Mitochondria, the prominent ancient organelles that emerged two billion years ago, are currently driving interests for their significant roles in the medical discipline [60]. There are a vast number of postulates about the involvement of mitochondria in several types of diseases; even some are still controversial. Mitochondria were previously known with many different terms like mitogel, interstitial bodies, and sarcosomes as reviewed back in 1918. It was initially discovered in 1888, and mitochondria are membrane-bound organelles that swell in water [34]. Possessing their own genomic materials, this special organelle is believed to originate as a self-sufficient single-cell organism and closely resembled modern prokaryotes [61]. Structurally, mitochondria consist of double membranes (inner and outer membranes). The main role played by mitochondria is to be “the powerhouses of the cell” and to produce adenosine triphosphate (ATP), thus making their existence vital in the evolution process of complex organisms. Otherwise, modern eukaryote cells need to depend solely on anaerobic glycolysis for ATP production, about 15 times fewer than the complete metabolism process in mitochondria [62–64].

**Figure 2:**
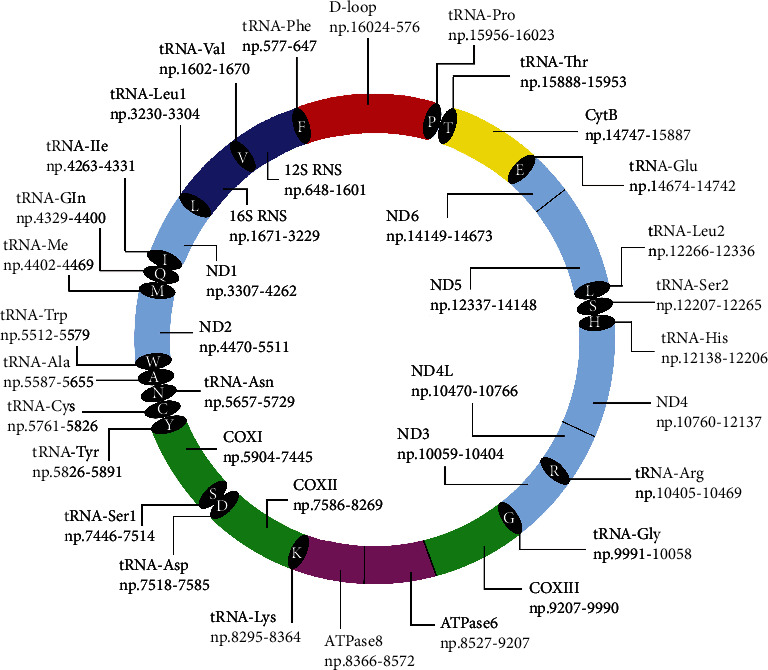
Schematic diagram of human mtDNA (16569 bp) with base pair range for each gene.

Human mitochondrial genomic material owns a closed circular shape and double-stranded, consisted of 16569 nucleotide base pairs. An mtDNA comprises of 37 genes that encoded 13 polypeptides mRNA essential for the oxidative phosphorylation (OXPHOS) system and mtDNA gene expression components (22 transfer RNAs and 2 ribosomal RNAs) [1, 63]. It was suggested that mtDNA is lacking repair enzymes for damage repairing activities and histone proteins for protection purposes. Thus, it is continuously exposed to oxidative agents that make mtDNA highly vulnerable to damage. It consequently leads to the accumulation of mtDNA mutations [63, 65, 66]. The schematic diagram of human mtDNA with base pairs range and nucleotide position (np) for each gene is shown in Figure 2.

### 4.2. Oncogenic Events Initiation by Mitochondrial Dysfunction and Membrane Potential Loss

Oncogenic occurrences in tumor cells proved to be linked with mitochondrial dysfunction through the retrograde pathway [54, 67]. According to Woo and colleagues, retrograde signaling is affected by respiration reduction and distinguishable from another pathway (intergenomic signaling pathway) that depends on mtDNA [68]. Their study shows that intergenomic signaling-targeted genes of *Saccharomyces cerevisiae* were downregulated in lacking mtDNA rho^0^ (mitochondrial depleted) cells, in contrast to lacking respiration rho^+^ (mtDNA existed) cells which suggested to affect nuclear genes expression [68]. Conclusively, mitochondrial dysfunction occurred when the mtDNA copy number reduced, therefore disrupting the *Δψ*m [31, 45].

Defective nuclear genes of mitochondrial biogenesis other than sustenance of mtDNA integrity and deoxynucleotide pools are postulated to cause mtDNA depletion [69]. The mtDNA copy number reduction was found responsible for mitochondrial genomic instability, leading to energy metabolism alteration that enhances tumor progression [67, 70]. Yang and Kim stated that several aggressive characteristics such as apoptosis, metabolic shift–glycolysis, and increased invasiveness in human cancers were correlated with reduced mtDNA copy number [32]. Alterations of genetic composition caused the metabolic shift–glycolysis changes, a crucial factor for tumor cell reprogramming [31]. Guha and colleagues had demonstrated the loss of invasiveness and induced cell reprogramming for epithelial–mesenchymal transition (EMT) in metastatic breast tumors [71]. In their study, the activation of calcineurin-dependent signaling was targeted as retrograde signaling pathway markers. They claimed it was a novel regulatory mechanism when low mtDNA content induced the initiation of breast cancer stem cells and EMT in human mammary epithelial cells [71].

Additionally, mutations of both genomes are able to induce mtDNA dysfunction by changing mitochondrial function, causing respiratory defects which leads to mtDNA content alterations, majorly in cancer cells [31, 72]. Hypothetically, mtDNA copy number decrement may initiate mitochondrial genomic instability, hence regulating energy metabolism that contributes to tumor onset [67]. mtDNA copy number reduction is highly attained resulting from nuclear and mtDNA mutations combining effects [73]. Despite this, intensified mtDNA copy number was observed in some cancers during tumor onset, particularly one of the features for malignant cells and aged cells. mtDNA mutations and damaged respiratory system caused mitochondrial metabolic 4defects that triggered an essential feedback mechanism to increase mtDNA biogenesis and replication [73]. However, Lee et al. concluded that mitochondrial genomic instability (4977 bp deletion) is not significantly correlated with mtDNA copy number reduction, and both events independently happened in cancer [74]. The POLG enzyme is responsible for causing multiple large-scale deletions besides depleting mtDNA, later upsetting the OXPHOS [75].

Gourlay et al. associated reducing *Δψ*m with actin dynamics of protein channels embedded in the mitochondrial membrane. They claimed that prolonged opening of channels leads to increasing released of ROS into the cytoplasm [76]. Nonetheless, another study rejected the postulate as ROS was not a mediator responsible for decreasing *Δψ*m [47]. ROS production reflected the optimal value of *Δψ*m exponentially, and high *Δψ*m leads to the significant generation of ROS by the respiratory chain of mitochondria [77, 78]. In the meantime, low *Δψ*m caused detrimental side effect because incapability of generating ATP induced oxidative stress, then initiated reductive stress [78, 79]. According to Xiao and Loscalzo, reductive stress is a condition where the endogenous oxidoreductase exceeds the limiting capacity from excessive build-up of reducing components such as NADH [80]. ROS is responsible as a signaling and cell growth stimulator; therefore, its inhibition is not a good choice of therapeutic strategy. Apparently, ROS inhibition shows intensified tumor cell survival by upregulating antioxidant pathways which neutralized ROS-related cytotoxicity (Figure 1) [81, 82].

These mitochondrial dysfunction evidences relating to oncogenic events confirm the Otto Warburg theory from eight decades ago, insisting that mitochondrial respiration defects are triggering aerobic glycolysis and cancer [83]. However, Zong et al. had pointed out that not all tumors showing the same aerobic glycolysis characteristic as the “Warburg theory”; in fact, tumor growth depends on mitochondrial functions which altogether eradicate mitochondrial dysfunction. Pathogenic mitochondrial genome mutations are known to enhance cancer cell proliferation, but selective pressure in many tumor cells allows retention of functional mitochondria. Nevertheless, accumulation of functional mitochondria is able to sustain malignant growth of some tumors; thus, mtDNA elimination is believed to limit tumorigenesis [84].

### 4.3. Mitoepigenetics Involvement in Cancer

The birth of mitoepigenetics started with the first discovery of mtDNA methylation, recorded back in 1970s using radiolabeling [85]. Recently, the mitoepigenetics field has largely driven attention for new investigations which accommodate researchers with new data and findings, especially in association with cancer. Limited methodology availability has been the reason that this field is scarcely explored until a few years ago [86]. The term “mitoepigenetics” was originated from the contribution of epigenetic mechanisms towards the regulation of mtDNA transcriptions and replication [87]. Besides, mitoepigenetics are reported to modulate cell fate, cell cycle, physiological homeostasis, bioenergetics, and various pathologies [88]. Due to the fact that mitochondria are lacking of histones and CpG islands, with different structures to be compared with nuclear chromatin, mtDNA methylation is the most investigated area [89].

Assorted studies pointed out that mtDNA transcription and replication processes are related to mtDNA methylation that was discovered in the D-loop region. Evidences of methylation and hydroxymethylation presence in mtDNA genome had highlighted that mitoepigenetics are potentially participating in mitochondria impairment underlying cancer progression [86, 88], considering the well-known relatedness between mitochondria dysfunction and cancer initiation-cum-progression [82, 83]. TFAM posttranslational changes were suggested to be an important cancer progression regulator since positive correlations to multiple malignant cancers were found. Other than suggested to be crucially modulating the pathological processes of cancer, it is also possible that mitoepigenetics are the results of tumorigenesis [90]. This was shown by an in vivo study observing tumor formation in cancer cells without mitochondria, eliminating mitoepigenetics as the main factor of tumorigenesis [91].

## 5. mtDNA Biomarkers in Human Cancers

The interconnection between carcinogenesis and mitochondria was firstly proposed in 1973 by Schumacher and colleagues when they observed dissimilar mitochondrial structures in cancer patients from normal subjects [92]. Since then, there was a shooting up in the number of studies revolving in this particular topic with methodology advancement, using DNA scanning technologies to detect point mutations and deletions [93]. As compared to nDNA, mtDNA owns inadequate repair mechanisms and high susceptibility to mutations which proposed its involvement with carcinogenesis [94]. The mtDNA is beneficial as a biomarker for carcinogenic studies since it consists of 37 genes with lacking features like introns; thus, most mutations will occur in coding regions. These mutation accumulation suggested to harvest potential biological importance that leads to tumor formation [94]. Additionally, several other mtDNA advantages including its small size, easy to extract, no genetic rearrangements, and fast mutation rates would favor molecular researches. Besides, a high copy number of mtDNA (up to thousands of copies per cell) required only minimal tissue samples for analysis purposes of rare disease studies [95]. The minimal tissue amount needed would be a significant feature for developing mtDNA biomarkers in cancer as tumor biopsies typically available in small amount.

### 5.1. Large-Scale Deletions

According to Chen et al., large-scale deletions are commonly known to be responsible for mitochondrial diseases [96]. Listed, there are three types of large-scale deletions: class I (deletions occurred within two direct repeats of identical sequences), class II (imperfect repeats flanked the deletions), and class III (deletions flanked by no direct repeats). Hypothetically, these deletion generations resulted from slippage mispairing of two repeats during replication for class I/II, while class II/III occurred during repairing of mtDNA double-strand breaks. Although deletion occurrences were less frequent, it was believed to be the culprit for various diseases and cancers [96].

#### 5.1.1. 3.4 kb (3379 bp) Deletion

This mtDNA large deletion occurred between 10743 and 14125 np. It was patented by Parr et al. [97], providing established kit for breast and prostate cancer detection. The kit allows cancer detection by quantifying 3.4 kb deletion, and elevated amount indicates cancer existence in individuals [97]. According to Parr et al. [98], deletions detected in proximal benign tissue suggested early tumorigenesis or pending transformation into cancer cell possibility [98]. This is called field-effect or cancerization, an occurring event before tumorigenesis that recognized minute tumor foci existence using quantitative polymerase chain reaction (qPCR). It allows identification of proximal benign from malignant prostate tumor biopsies [99]. The deletion is highly beneficial in determining different prostate tissue types, either benign, malignant, or proximal to malignant [100].

Small- and large-scale deletion in prostate cancer would cause functional cellular mtDNA reduction [101]. Creed et al. [102] proved the deletion as clinically significant in prostate cancer and suggested it as an accurate cancer predictor with 100% sensitivity and 90% specificity. Another study suggested the 3.4 kb deletion biomarker and mtDNA copy number combination for a better prostate cancer determination. Possible sample types consisted of formalin-fixed paraffin-embedded (FFPE) tissues, urine, and serum from patients with and without prostate cancer [103]. Additionally, 3.4 kb deletion was recommended as a prostate tissue-specific biomarker for its ubiquitous presence in cancerous prostate [104]. Prostate Core Mitomic Test (PCMT), a 3.4 kb deletion commercial kit, was used in a study involving USA multicentre prostate cancer patients [105]. Initially, 644 patients tested with the kit were negative, and only 35 patients were rebiopsied (five false-negative cases). Therefore, the kit was suggested as clinicians' decision-making aid for rebiopsy to reduce the cost [105].

#### 5.1.2. 4977 bp Deletion (mtDNA^4977^)

Initially reported in neuromuscular disease Kearns–Sayre syndrome in 1989 and cancer studies afterward, in this large-scale class I deletion, mtDNA^4977^ is primarily associated with aging. It is a common deletion with missing mtDNA nucleotide sequences starting at 8470 to 13447 np [106]. A published patent provides methodologies to control the mtDNA^4977^ rate in mitochondria [107]. The methods emphasized on cellular work by modulating sirtuin activity meant for drug testing, stem cell production, or curing age-related diseases and uncontrolled growth. A comprehensive overview regarding mtDNA^4977^ in human cancers was previously discussed [1]. mtDNA^4977^ is a well-known biomarker with huge establishment in various cancer studies worldwide.

mtDNA^4977^ association with breast cancer has been proven by a large study in China involving blood and tissue samples from 107 breast carcinoma and 118 benign breast disease patients [108]. The mtDNA^4977^ rate was significantly higher in breast carcinoma patients' blood compared to a benign-type disease, adjacent tissues, and healthy controls. This suggested mtDNA^4977^ as a potential noninvasive biomarker for breast cancer detection [108]. Another study from Argentina showed a higher mtDNA^4977^ in control samples, suggesting other underlying mechanisms (besides normal aging) are regulating the variant accumulation in breast cancers [109]. Similarly found in Vietnam, researchers postulated that mtDNA^4977^ is a common event for breast cancer with close association to oestrogen receptor-positive patients [110].

Colorectal cancer study observed similar trends, with reported mtDNA^4977^ as high as 92.4% in the Swedish adjacent tumoral tissues [111]. In contrary, Chen et al. [112] reported 16.3% common deletion in cancerous tissues compared to 12.5% in adjacent tissues. Dani et al. [113] recruited gastric carcinoma samples, and all adjacent tissues accumulated mtDNA^4977^, a higher proportion than cancerous tissues. However, another study from China demonstrated contradicting results with a higher mtDNA^4977^ rate (79.6%) in cancerous tissues [114]. mtDNA^4977^ was initially studied in Japanese population for hepatocellular carcinoma [115]. Afterwards, mtDNA^4977^ was claimed responsible for hepatocellular carcinoma development and progression [116]. Betel quid chewing was correlated with increased mtDNA mutations, thus contributed to oral carcinogenesis with a coexisting participation factor, cytochrome P450 2E1 gene polymorphism [117, 118].

Recently, mtDNA^4977^ was screened in Malaysian population with brain tumors, and 32% showed deletions in cancerous glioma and meningioma tissues, but none in control samples [119]. The finding was supported by a study on hepatocellular carcinoma which demonstrated mtDNA^4977^ only in cancerous tissue [120]. Hypothetically, noncancerous tissues' lower rate was correlated with cancer stage, significant mtDNA content rate, and increased ROS content from decreased antioxidative activities [112, 114, 120]. mtDNA^4977^ screening were also conducted in various types of cancer: the skin, lung, and endometria [121–123], showing higher frequency in adjacent tissues. These results might suggest the cancerization effect, similarly to 3.4 kb deletion. Overall, the mtDNA^4977^ accumulation might be multifactorial and possibly affected by external environmental factors, genetic predisposition, and ethnicity.

#### 5.1.3. Other Deletions

There are several other large-scale deletions associated with cancers. For example, mtDNA 3895 bp deletion was patented in 2011 for early cancer detection cum diagnosis of nonmelanoma skin cancer and sun exposure. It was observed at the minor arc of mtDNA with the range spanning from 547 to 4443, starting at the D-loop mtTF1-binding site till tRNA methionine [124]. The same deletion was firstly described in 1991 by Moraes et al., in two of their patients with progressive external ophthalmoplegia. However, the frequency of detection was 10 times less frequent than mtDNA^4977^ [125]. Afterward, a study involved 104 age-matched subjects who presented a significantly higher frequency of 3895 bp shown by a “usually” sun-exposed skin with predominantly nonmelanoma skin cancer. It was stated that simultaneous screening of mtDNA^4977^ in a similar population was 50% lower than the 3895 bp deletion [126]. The findings were further confirmed using the qPCR analysis, and same results were acquired, a greater level of 3895 bp deletion in the usually sun-exposed skin than the occasional sun-exposed group [127].

Another significant large-scale deletion is the 4576 bp deletion reported by Zhu and colleagues in 2004 [128]. The deletion was suggested as an indicator for breast cancer as referred to their results. Their study involved 39 breast cancer patients, as high as 77% of breast cancer tissues discovered with 4576 bp deletion while 13% were found in adjacent tissues. As reported, the same deletion was not observed in 23 normal patients (without breast cancer) which rationalized 4576 bp deletion as a breast cancer screening tool even in samples with mixed or low cellularity [128].

### 5.2. mtDNA Copy Number

The mtDNA copy number (mtDNA content) referring to the contents of the mitochondrial genome in each cell, approximately 10^3^ to 10^4^ copies, depends on types of cells and developmental stage [129]. Hypothetically, mtDNA content increment or reduction was likely to be cancer-specific. The content deviation is naturally affected by cell-specific energy requirements besides responding towards physiological signals and conditions [130]. Dai et al. supported that energy metabolism and aerobic ATP production determined the mtDNA content [131]. As discussed earlier, mtDNA copy number changes may lead to mitochondrial instability and regulate energy metabolism, yet initiate tumorigenesis. Thus, mtDNA biogenesis deficit in cancer cells may give rise to mtDNA copy number reduction as seen in various solid tumor studies. This condition was frequently correlated with reduced OXPHOS protein levels, tumor aggressiveness, and clinicopathologic parameters in different cancer types [131].

The mtDNA copy number is closely related to mtDNA mutations when involving the vital region. In hepatocellular carcinoma, a study concluded that reduced mtDNA content was correlated to mitochondrial biogenesis impairment with mutations in D-loop region [132]. This is supported by a work from Lee and coworkers, as 61% of hepatocellular carcinoma patients with mtDNA D-loop mutations demonstrated reduced mtDNA content [133]. The D-loop plays a crucial role as major control sites for mtDNA transcription and replication; thus, any mutations in this hotspot results in modification of nuclear protein binding affinities, leading to mtDNA content reduction [1, 27, 28]. Another study which involved breast cancer patients also suggested D-loop mutations as a contributor for mtDNA copy number reduction [134]. Previous large-scale deletion studies with different sources of samples such as tissues or liquid biopsies (blood, saliva, or urine) also reported the abundance of mtDNA in cancer cells using qPCR analysis [103, 108, 112, 120, 132].

Associations were made between mtDNA copy number and breast cancer risk, development, and neoplastic transformation [134–136]. Guha and colleagues demonstrated that mtDNA copy number reduction significantly generated breast cancer stem cells and induced metastatic characteristics [71]. Variable mtDNA content was altered by genomic heterogeneity of particular cancer, as shown in prostate and colorectal cancer [137, 138]. Previously, mtDNA copy numbers were found comparably increased and decreased in colorectal cancer. Reported as risk and/or prognosis evaluation tools of cancer detection, some studies have shown contradicting findings [138]. For example, a lower mtDNA copy number suggested to reduce 3-year survival and correlated with lymph node metastasis [139]. Another research proposed that overall survival and relapse-free survival worsen with increased mtDNA copy number [140].

Additionally, mtDNA copy number changes determined chemotherapy response and play its role as a predictive biomarker [75]. Hsu et al. supported the statement through their research on anthracycline-containing treatment among breast cancer patients. Their findings demonstrated higher chances of disease-free survival in breast cancer patients with a lower mtDNA copy number [141]. Additionally, Yu et al. mentioned that mtDNA depletion was correlated with a lower chance of disease-free survival and higher tumor grades. Besides, the mtDNA content in tumor tissues showed significantly decreasing mtDNA count as compared to normal adjacent tissues [134].

### 5.3. Circulating Cell-Free mtDNA (cf-mtDNA)

Cell-free mtDNA is defined as leaking cellular mtDNA from within mitochondria into the cytosol or peripheral blood circulation, caused by disruption of the normal mitochondrial life cycle (mtDNA replication and replacement), with compromised mitophagy process of damaged mtDNA [142, 143]. Featuring the ancestral prokaryotic characteristic of unmethylated CpG dinucleotides, mitophagy-escaping mtDNA was believed to be a major activator of the Toll-like receptor 9 (TLR9) pathway that consequently causes downstream inflammation response [142–144]. The interconnection between mtDNA and TLR9 was initially discovered in 2010 [145]. In this discovery, the cf-mtDNA in the blood triggered TLR9 on neutrophils during the systemic inflammatory response syndrome [145]. Interestingly, Singh and coauthors stated that chronic inflammatory response regularly provided a favorable environment for cancer development through cell mutation and proliferation [146]. Although it is a controversial diagnostic tool, the circulating levels of mtDNA had been used to diagnosed cancer and sepsis [147]. The authors also suggested cf-mtDNA as a biomarker for detecting individuals with metabolic syndrome or predicting the risk of future diabetic development [147]. cf-mtDNA holds a great potential as a biomarker since metabolic syndrome was highly correlated to increased risk of common cancers. The correlation was previously reported in a meta-analysis study, which included 116 datasets (38940 cancer cases) extracted from 43 different articles [148].

Similarly released into blood circulation, cell-free nDNA (cf-nDNA) was recommended as noninvasive liquid biopsy since higher levels of cf-nDNA were previously reported in cancer patients, as compared to healthy controls [149]. The wide range level of cf-nDNA (between 0.01% to more than 90%) correlates with several clinical features such as tumor burden and therapy response [150]. However, since total nDNA concentration is very low in body fluid samples, it is more conducive to screen cf-mtDNA because of a higher mtDNA copy number, simpler structure, and shorter length [151]. Naturally alike, vigorous researches were conducted to find the potential link between cf-mtDNA and various cancers. cf-mtDNA is much more preferable as a noninvasive biomarker with easier sampling procedures and handling during molecular analysis [19, 102, 108, 135, 136, 140, 152, 153].

A study suggested that mtDNA content analysis from peripheral blood could serve as a noninvasive biomarker and predictor for hepatocellular carcinoma risk in patients with hepatitis C [152]. Supported by other authors, their study used plasma and found it as a promising complementary sample alongside tissue specimens that served as a predictor for diagnosis and prognosis of lung cancer [154]. Several studies conducted in head and neck cancer areas showed significantly higher cf-mtDNA levels in cancer patients to be compared with control samples [155–157]. The cf-mtDNA levels were found to be increasing with progression of cancer, associated with lymph node metastasis, and predictive with the survival of cancer patients [156]. Meanwhile, a recent study by Kumar and colleagues proposed that cf-mtDNA is potentially fit as a diagnostic biomarker of head and neck cancer. This is due to its high association with smoke and smokeless tobacco, alcohol, and betel quid chewing [157].

Analyzed cf-mtDNA as diagnostic and prognostic biomarker in epithelial ovarian cancer showed significantly higher levels in cancer patients than healthy controls [158, 159]. However, only Meng et al. discovered the association between elevated cf-mtDNA levels and cancer progression cum poor prognosis [159]. cf-mtDNA was evaluated as biomarkers in several other studies of various cancer types such as endometrial cancer [160], prostate cancer [161], and glioma [162], albeit contradicting and inconclusive results regarding cf-mtDNA studies were previously reported, for example in breast cancer studies. cf-mtDNA content showed lower value in cancer patients than control samples in some research [163, 164], in contrast to other studies [135, 136, 165]. These contradicting results may suggest that the mtDNA copy number relationship with breast carcinogenesis is controlled by an underlying mechanism and remains unclear.

However, data consistencies depended highly on the normalization of methodologies, hence crossing out the possible causes of inconsistency which ensure reliable findings for future reference [166]. cf-mtDNA quantification using qPCR was expressed per volume of sample, unlike cellular mtDNA copy number measurement that used nuclear gene target for normalizing [167]. Different normalizing methods among studies might be the major contributor to data inconsistencies, as some reported cf-mtDNA copies per microliter of the sample, relative values from study groupings, and genomic equivalents per sample volume [157, 168, 169]. Moreover, the source of mtDNA content (whether cellular mtDNA or cf-mtDNA) obtained from the collected blood surfaced an issue of the value that could not be determined using the current methods. As such, Rosa et al. have recently proposed the absolute quantification method to differentiate the mtDNA content source by dividing the whole blood into several fractions (whole blood, peripheral blood mononuclear cells, plasma, and serum) [167]. The authors compared the mtDNA content in diabetic patients from healthy controls and found that the cf-mtDNA content was twofold higher in plasma and serum. Thus, it shows the significance of measuring cf-mtDNA alteration as another important data to consider [167].

### 5.4. Mitochondrial Microsatellite Instability (mtMSI)

Microsatellites are short tandem repeats (mononucleotide or dinucleotide) from 1 to 6 bp which scattered all over not only in nuclear but also in mitochondrial genome [170]. The variations include deletions or insertions, able to cause frameshift mutations [171]. Suggested by Bendall and Sykes [172], DNA polymerase *γ* slippage and its poor fidelity elevated the error-prone mtDNA replication process. DNA polymerase *γ* acted as an oxidative damage target, and when badly impaired, it may lead to extensive mtDNA replication and repair errors [173]. Besides, mammalian mitochondrion has its own inefficient mismatch repair system, in which any defects may cause mtMSI formation [174]. Mitochondrion lacks mtDNA repairing genes; thus, all protein components are nuclear-encoded and imported (intergenomic cross talk) for mitochondrial genomic integrity maintenance [175]. However, responsible mechanisms involving mtMSI are not much known [174].

Among all, the most frequently reported mtMSI is located in the D-loop region [176]. The D310 site is a mutational hotspot in primary tumors and described as a highly polymorphic homopolymeric C stretch [177]. The D310 site, a mutational hotspot in primary tumors, was described as a highly polymorphic homopolymeric C stretch. According to Sanchez-Cespedes et al. who cited Xu and Clayton in 1995, its location in hypervariable region II (HVRII), 92 bp from replication origin (heavy strand), is involved in R-loop formation––a stable RNA-DNA hybrid which triggered mtDNA replication [177]. The authors suggested D310 as a new cancer detection tool [178]. D310 alteration was claimed as the initial event in malignancy with the potential to be an early premalignant cancer marker [75]. Two different studies demonstrated the D310 detection in normal adjacent epithelial cells of breast [179] and gallbladder carcinomas [180], in conjunction with a mutation-carrying cancerous tissues. The D310 mtMSI was also observed in 12% of brain tumor patients [176], 34% in rectal carcinoma, and 38% in sigmoid or colorectal carcinoma [181].

Another potential marker and a common mtMSI is D16184, with a similar wild-type structure to D310 (homopolymeric C stretch with T nucleotide interruption) [174]. The D16184 was located in the hypervariable region I (HVRI), in proximity to the 3′-end of termination-associated sequence within the 7S DNA binding site, thus vital for mtDNA biogenesis [182]. A vast number of studies reported the presence of D16184 involving various cancer types such as gastric (16.1%) [183] and endometrial carcinoma (14%) [182], interestingly at high prevalence (70.3%) in a recent acute myeloid leukemia study [184].

### 5.5. Somatic mtDNA Alterations

Researchers tried to correlate somatic mtDNA alterations and cancer. Evidence showed that mtDNA changes play a role as a contributing factor, whether in the development or progression of cancer [65]. Neoplasm studies reported about 25 to 80% of somatic mtDNA mutations and believed to cause neoplastic transformation by shifting energy sources of cells, modulating apoptosis, and increasing oxidative stress [65]. Findings of significant variations in cancer pursued after Polyak and colleagues discovered alterations in primary tumors of colorectal cancer, since potential consequences of abnormal metabolic and apoptotic processes in cancer were found related to homoplasmic mutations [185].

Abundant studies were conducted for mtDNA A12308G alteration, located in the variable loop next to the anticodon stem of tRNA^Leu (CUN)^ [186]. Mitochondrial tRNALeu (CUN) largely encoded proteins for the respiratory chain, while nucleotide at position 12308 involves in tertiary interaction. Thus, any changes in this position are believed to affect respiratory chain synthesis and tertiary structure [187]. It was firstly discovered by Houshmand et al., who introduced it as common polymorphism and nonpathogenic [188]. However, an mtDNA haplotype study suggested A12308G as a mitochondrial predisposition factor to prostate and renal cancer in North American white individuals [189]. In Poland, a breast cancer study detected the same alteration in 12% of studied population. The variant was closely associated with cancer cells and neoplastic process [187]. According to Mohammed and coauthors, A12308G is a potential diagnostic tool for colorectal cancer and considered pathogenic in combination with other mtDNA alterations [190]. A study among the Indian population supported that A12308G increased the risk of oral cancer, similarly displayed by another locus, A10398G [186].

Likewise, Covarrubias et al. reported similar findings by detecting both alterations (A12308G and A10398G) in their study, subsequently proposed to increase the risk of breast cancer development [191]. However, the association between A10398G alteration and increasing risk of breast cancer was debated due to conflicting results and haplotype grouping [192]. Salas and coauthors analyzed the inconsistencies and conclusively stated that most case-control association studies were slightly undertaken with disputed scientific standards. Therefore, complex and multifactorial diseases with unclear underlying mechanisms should be deeply studied to prevent false-positive conclusions [192]. Despite the conflicts, screening of A10398G continued in other cancers like tongue squamous cell carcinoma [193], although it was suggested as a poor prognosis marker for non-small cell lung cancer [194].

### 5.6. mtDNA and Mitochondrial RNA (mtRNA) Methylation

DNA methylation modification was said to be the most investigated mechanism in mitochondria. Meanwhile, it was reported that the impairment of mtDNA methylation patterns could be influenced by nDNA genomic changes other than environmental factors [86]. Various studies reported different sources of mtDNA methylation-positive regulators such as maternal smoking [195], high glucose [196], and lipid levels associated with dietary intake [197].

In recent years, several conducted researches observed positive relationship between impaired mtDNA methylation and cancer. An in vitro study detected higher levels of CpG and non-CpG (CpH) in liver cancer cells using bisulphite sequencing when compared to nontumorous cells, whereas the finding was totally different for breast cancer cells with higher percentage that was detected in normal cells. It was claimed that methylation patterns are cell-type specific [198]. Another study tested peripheral blood collected from five different families where one breast cancer patient had positively correlated D-loop methylation with breast cancer risk. The authors also suggested that the D-loop region displayed familial-specific methylation pattern, and it was maternally inherited [199]. Newly reported, higher CpG and CpH mtDNA methylation levels were discovered in head and neck cancer tissues to be compared with noncancerous, using the nanopore sequencing method. This current method was suggested to be a useful tool for sequencing mtDNA bases modification since it prevents bisulphite and PCR amplification bias [200].

Sun et al. had described the mechanism which relates mtDNA methylation and tumorigenesis in their cellular models' study, while reporting the negatively associated results with mtDNA transcription. Decreasing 5mC levels during tumor progression of glioblastoma and osteosarcoma cells were detected at mtDNA-specific sites, which potentially conform to the increasing level of the mtDNA copy number. Later on, the 5mC levels would also increase to inhibit further mtDNA replication process, implying that the sufficient mtDNA number had been restored for tumorigenesis initiation. The authors claimed that a lower mtDNA copy number exhibited by cancer cells was due to a “pseudodifferentiated” state [201]. Low mtDNA methylation was also reported in studies that tested cervix cancer and adenomas samples [202, 203]. It was suggested that the decreased level of 5mC during tumorigenesis might be a potential prognostic marker for cancers [90].

Another part of methylation implies the crucial role of posttranscriptional modulations for RNA processing, which happens when mtDNA is transcribed using continuous polycistrons into RNA [90]. Stewart et al. discovered the diverse accumulation of mtRNA transcripts across human cancers [204]. It was supported by a study involving 12 different cancer types, showing remarkable alterations in mitochondrial m^1^A and m^1^G tRNA methylation levels [205], thus suggesting to significantly affect mitochondrion-mediated metabolism [206]. The changes predicted the poor prognosis of patients with kidney renal clear cell carcinoma [205]. In an in vitro study for cisplatin sensitivity testing in oral squamous cell carcinoma cell line, the MT-CO1 and MT-CYB genes were found hypermethylated with concomitant high expression levels. It was claimed that mtDNA methylation enhanced genes expression, implying to affect posttranscriptional modifications of polycistronic mitochondrial mRNAs [207].  Table 1 summarizes the protruding and potential mtDNA biomarkers in various human cancer studies.

## 6. Future Perspectives of mtDNA Biomarkers

Mitochondrial interventions and gene therapy were briefly reviewed and associated with mitochondrial diseases [208–210]. Meanwhile, mitochondrial biomarkers may serve as an early detection tool through the development of a commercial kit (PCMT) as previously discussed [105]. However, future treatment improvement could be useful for treating asymptomatic cancer, in which symptoms development usually occurred in later stages with increasing severity, leading to limited options of treatment [211]. Considering the evidences of mitochondrial dysfunction and significant relativity to carcinogenesis, gene therapy development or other mitochondrial interventions are relevant as a potential tool for cancer therapeutics.

Researchers can identify the pathogenicity and therapeutic potential of a particular mtDNA mutation due to current advancements in vitro mitochondrial intervention. Previously, the patented method proposed a procedure of transferring artificial healthy mitochondria which removes damaged mtDNA without genetic manipulation [210]. A similar concept has been described by Caicedo et al. developed MitoCeption, a tool for evaluating the effects on cell metabolism and function by transferring mitochondria from mesenchymal stem/stromal cell into the cancer cell. In consequence, it allows a deeper understanding of cancer cell metabolic reprogramming which significantly correlated with tumor progression and anticancer drug resistance [212].

Another method is the mtDNA replacement which enables mtDNA heteroplasmic ratio shifting through existing mtDNA repairing or nonnative mtDNA production, done by targeting specific mtDNA sequences [210]. This was achieved through mtDNA gene editing––a double-strand break repair system. This inefficient system emphasized the degradation of pathogenic mtDNA by introducing endonucleases (zinc finger nucleases (ZFN) [213] and mitochondrially targeted transcription activator-like effector nucleases (mitoTALENs) [214]), thus substituting with wild-type mtDNA [210]. However, the mtDNA replacement method was disputed due to bioethical issue, since it involves mtDNA manipulation. Unlike nuclear genome modification, modified germline mtDNA was inherited to offspring, hence disturbing future genetic pool [215, 216].

## 7. Conclusion

Comprehensive insights on biomarkers have been thoroughly discussed in this review, hoping to provide an in-depth understanding of mitochondrial correlation to carcinogenesis. Fascinatingly, intergenomic cross talk between nucleus and mitochondria (anterograde, retrograde, and in-between pathways) is the turning point that challenged researchers to discover evidences regarding mtDNA alterations and dysfunction effects in driving cancer development and progression. mtDNA will be a unique target for cancer treatment as it is not strictly controlled by cell cycle [94]. Moreover, several mtDNA features such as high mutations susceptibility, easy to extract, and minimal tumor tissue requirements would be advantageous for cancer researches, including the rare cancer types.

Cancer research is a broad area, which accommodates researchers with opportunities for making new discoveries, especially in the mitoepigenetics field. Little is known, but mitoepigenetics' role in regulating mitochondria functions should not be taken for granted. This might lead to new options for cancer therapy strategies, which initially calls for vast numbers of studies to validate its association to cancer. Carcinogenesis is a complex process with multifactorial contributors, leading to tumor initiation, growth, progression, and therapeutic possibilities. Hence, many different aspects need to be comprehended, whether in nuclear or mitochondria. Compiled references and information in this review might provide clear perspectives for future investigations, especially in mtDNA biomarker studies. Recent successes in mitochondrial intervention and gene therapy for mitochondrial diseases could be the benchmark for further discoveries of potential mtDNA biomarkers with high specificity and sensitivity. Thus, a great future holds for further advancement in cancer research and new therapeutic strategies development.

## Figures and Tables

**Table 1:** Summary of protruding and potential mitochondrial DNA biomarkers in various human cancers.

mtDNA biomarkers	Human cancers	Findings	References
Large-scale deletions			
3.4 kb (3379 bp)	Breast and prostate	(i) Patented kit for cancer detection, Prostate Core Mitomic Test kit; deletion was detected in proximal benign tissues (field-effect or cancerization), suggesting early tumorigenesis. (ii) Deletion was suggested as cancer predictor with 100% sensitivity and 90% specificity.	[97–100, 102]
4977 bp	Breast	(i) Deletion was higher in cancer patients, suggesting it as a potential noninvasive biomarker for breast cancer detection (China). (ii) Higher deletion in control samples than cancerous tissues (Argentina, Vietnam).	[108–110]
	Colorectal; gastric	(i) Higher deletion in control samples than cancerous tissues (Sweden; Brazil). (ii) Deletion was higher in cancerous tissues (China).	[111, 113, 112, 114]
	Hepatocellular	(i) First mtDNA^4977^ study, with higher detection in adjacent tissues (Japan). (ii) Common deletion was responsible for cancer development and progression.	[115, 116]
	Oral	(i) Associated betel quid chewing with increased mtDNA mutations, suggesting cytochrome P450 2E1 gene polymorphism as coexist factor.	[117, 118]
	Brain; hepatocellular	(i) Deletion was detected only in cancerous tissues (Malaysia; China).	[119, 120]
	Skin; lung; endometrial	(i) Higher deletion detected in adjacent tissues (Germany; China; Poland).	[121–123]
3895 bp	Skin	(i) Deletion was patented in 2011 for cancer detection and diagnosis. (ii) Higher deletion frequency in sun-exposed skin with predominant nonmelanoma cancer; mtDNA^4977^ detection was 50% lower.	[124, 126, 127]
4576 bp	Breast	(i) Deletion in 77% of cancerous tissues with no deletion in normal subjects; suggested as breast cancer screening tool.	[128]

mtDNA copy number	Hepatocellular; breast	(i) Reduced copy number was correlated to D-loop mutations.	[132–134]
	Breast	(i) Associated copy number with breast cancer risk, development, and neoplastic transformation. (ii) Reduced copy number significantly induced breast cancer stem cells and metastatic characteristics. (iii) Copy number changes determined chemotherapy response; low copy number shows higher chances of disease-free survival. (iv) mtDNA depletion correlated with lower chances of disease-free survival and higher tumor grades.	[134–136, 71, 141, 134]
	Prostate; colorectal	(i) Genomic heterogeneity altered mtDNA content.	[137, 138]
	Colorectal	(i) Low copy number reduced 3-year survival and correlated with lymph node metastasis. (ii) Increased copy number worsen the overall survival and relapse-free survival.	[139, 140]

Circulating cell-free mtDNA (cf-mtDNA)	Hepatocellular	(i) Suggested as noninvasive biomarker and predictor for cancer risk in hepatitis C patients.	[152]
	Lung	(i) Predictor for diagnosis and prognosis of cancer.	[154]
	Head and neck	(i) Significantly higher levels observed in cancer patients than controls; increased levels with cancer progression, associated with lymph node metastasis and predictive with cancer survival; suggested as diagnostic biomarker due to high association with smoke and smokeless tobacco, alcohol, and betel quid chewing.	[155–157]
	Epithelial ovarian	(i) Showed significantly higher levels in cancer patients which was suggested as diagnostic and prognostic biomarker.	[158, 159]
	Endometrial; prostate; brain	(i) Evaluated as a biomarker.	[160–162]
	Breast	(i) Lower levels in cancerous samples than controls. (ii) Higher levels in cancerous samples than controls.	[163, 164, 135, 136, 165]

mtMSI			
D310	Breast; gallbladder	(i) Mutations detected in both cancerous and normal adjacent tissues.	[179, 180]
	Brain; rectal and colorectal; tongue squamous cell	(i) Detected in cancerous patients (12%, 34%, 38%, and 25%).	[176, 181, 193]
D16184	Gastric; endometrial; acute myeloid leukemia	(i) Detected in cancerous patients (16.1%, 14%, and 70.3%)	[183–184]

Somatic mtDNA alterations			
A12308G, tRNA^Leu (CUN)^	Prostate and renal	(i) Mitochondrial predisposition factor in North American white individuals.	[189]
	Breast	(i) 12% changes detected among studied population in Poland and closely associated with neoplastic process. (ii) Increased the risk of cancer development.	[187, 191]
	Colorectal	(i) Potential diagnostic tool for cancer and pathogenic when combined with other mtDNA alterations.	[190]
	Oral	(i) Increased the risk of cancer.	[186]
A10398G	Oral	(i) Increased the risk of cancer.	[186]
	Breast	(i) Increased the risk of cancer development.	[191]
	Tongue squamous cell	(i) Detected in 62.5% of cancerous tissues.	[193]
	Non-small cell lung	(i) Suggested as a poor prognosis marker.	[194]

Methylation			
mtDNA	Liver and breast	(i) Bisulphite sequencing detected higher levels of CpG and non-CpG in liver cancerous cell lines compare to noncancerous, while higher levels in normal cells than breast cancerous cells.	[198]
	Breast	(i) Positively correlated D-loop methylation with cancer risk which is maternally inherited; displayed familial-specific methylation pattern.	[199]
	Head and neck	(i) Higher CpG and CpH levels detected in cancerous tissues than noncancerous using nanopore sequencing that prevents bisulphite and PCR bias.	[200]
	Glioblastoma and osteosarcoma	(i) Decreased 5mC levels detected while mtDNA copy number increased which regulates transcription process.	[91]
	Cervix; adenoma	(i) Low mtDNA methylation detected in cancerous tissues.	[202, 203]
mtRNA	Kidney renal clear cell	(i) Predicted poor prognosis of cancer patients.	[205]
	Oral squamous cell	(i) Observed hypermethylation of MT-COI and MT-CYB with concomitant high expression levels in cancer cell lines.	[207]

mtDNA: mitochondrial DNA; mtRNA: mitochondrial DNA; cf-mtDNA: circulating cell-free; mtMSI: mitochondrial microsatellite instability.

## Data Availability

The data supporting this review are from previously reported studies and datasets, which have been cited.
